# Evidence that the second human pegivirus (HPgV-2) is primarily a lymphotropic virus and can replicate independent of HCV replication

**DOI:** 10.1080/22221751.2020.1730247

**Published:** 2020-02-26

**Authors:** Zhengwei Wan, Junwei Liu, Fengyu Hu, Jingwei Shui, Linghua Li, Haiying Wang, Xiaoping Tang, Chengguang Hu, Yuanhao Liang, Yuanping Zhou, Weiping Cai, Shixing Tang

**Affiliations:** a Department of Epidemiology, Guangdong Provincial Key Laboratory of Tropical Disease Research, School of Public Health, Southern Medical University, Guangzhou, People’s Republic of China; b Department of Infectious Diseases, Nanfang Hospital, Southern Medical University, Guangzhou, People’s Republic of China; c Infectious Disease Center, Guangzhou Eighth People’s Hospital, Guangzhou Medical University, Guangzhou, People’s Republic of China; d Dermatology Hospital, Southern Medical University, Guangzhou, People’s Republic of China

**Keywords:** HPgV-2, persistent infection, resolved infection, lymphotropic, HCV

## Abstract

The second human pegivirus HPgV-2 is a novel blood-borne virus that is strongly associated with the hepatitis C virus (HCV) infection. However, the molecular evidence for their association as well as the natural history and tissue tropism of HPgV-2 remain to be elucidated. In this longitudinal study, a total of 753 patients including 512 HIV-1 and HCV co-infected patients were enrolled to characterize the natural history of HPgV-2 infection. Peripheral blood mononuclear cells (PBMCs) and liver biopsies were collected to determine the tissue tropism of HPgV-2 using immunohistochemical staining of the HPgV-2 antigen and in situ hybridization of HPgV-2 RNA. We documented both persistent HPgV-2 infection with the presence of HPgV-2 viral RNA and antibodies up to 4.6 years and resolved HPgV-2 infection, accompanied by a simultaneous decline of anti-HPgV-2 antibodies and clearance of HPgV-2 viremia. Furthermore, we observed the clearance of HCV, but not HPgV-2, by treatment with direct-acting antivirals (DAAs). Biochemical tests and pathological analyses did not reveal any indication of hepatic impairment caused by HPgV-2. HPgV-2 RNA and nonstructural antigen were detected in the lymphocytes, but not in the hepatocytes present in the liver biopsy samples. In addition, both positive- and negative-strand HPgV-2 RNAs were detected in PBMCs, especially in B cells. The present study is the first to provide evidence that HPgV-2 is a lymphotropic, but not a hepatotropic virus and that HPgV-2 replication is independent of HCV viremia. These new findings let us gain insights into the evolution and persistent infection of RNA viruses in humans.

## Introduction

Since the discovery of the first human pegivirus (HPgV) in 1995 [1], a growing number of pegiviruses have been identified in a wide range of hosts [1–7]. These positive-sense, single-stranded RNA viruses have recently been classified into eleven species (Pegivirus A-K) within the pegivirus genus of the Flaviviridae family [8]. Among them, two pegiviruses are known to infect humans, i.e. HPgV, formerly called hepatitis G virus (HGV) [9] or GB virus type C (GBV-C) [1], and the second human pegivirus (HPgV-2) [10] or human hepegivirus-1 (HHpgV-1) [5]. HPgV-2 was first reported in the US in 2015 [5, 10], and has since been documented in the UK, China, Vietnam, Cameroon, and Iran [11–16].

HPgV is believed to be a lymphotropic, but not a hepatotropic virus [17, 18], and can persistently infect humans without illness [19]. However, recent meta-analysis as well as several large studies all support a positive association of HPgV viremia with lymphoma [20–23]. Interestingly, there is some relevant information on pegivirus infection in animals. A pegivirus identified in horses appears to have been associated with an outbreak of equine serum hepatitis in the US [3]. Additionally, in 2016, a porcine pegivirus (PPgV) was shown to cause persistent infection and to generate histopathogenic changes in the liver [2]. These results suggested that PPgV may be hepatotropic and/or lymphotropic [24].

Previous studies revealed a low prevalence of HPgV-2 viremia in the general population, and an increased prevalence in patients infected with hepatitis C virus (HCV) [10, 12, 13, 15] as well as in people who inject drugs (PWID) and are co-infected with HCV and human immunodeficiency virus type one (HIV-1) [11, 15, 25, 26]. These data show a strong association between HPgV-2 and HCV and especially HCV/HIV-1 co-infection [10–13, 15, 25, 26]. Furthermore, HPgV-2 shares some common features with HCV, such as a type IV internal ribosome entry site in the 5’ untranslated region (5’ UTR) and a highly glycosylated E2 protein [5]. In view of these results, one may ask: (a) is HPgV-2 a hepatotropic virus that can cause liver disease? (b) is HPgV-2 replication dependent on HCV infection and replication? Detailed characterization of the newly discovered HCV- and HPgV-like virus could contribute to our understanding of the origin of HPgV-2 and the interaction between HPgV-2 and HCV, thereby enhancing our ability to study pathogenesis and immune responses that may be caused by HPgV-2 infection.

In this study, for the first time, we report that HPgV-2 infects peripheral blood mononuclear cells (PBMCs), specifically B-lymphocytes, but not hepatocytes. These results indicate that HPgV-2 is a lymphotropic and not a hepatotropic virus, which in turn may explain the lack of association with liver disease. Furthermore, we find that direct acting antivirals (DAAs) that specifically target and clear HCV could not inhibit HPgV-2 replication and that in DAAs-treated HCV patients, HPgV-2 replication was not correlated with HCV viremia. These new findings point to a direction for future clinical study targeting a possible association between HPgV-2 infection and lymphoma or B cell-related diseases. Moreover, our data provide clues for the resolution of the difference between the epidemiological association of HPgV-2 and HCV infection and the independence of HPgV-2 replication and HCV viremia.

## Materials and methods

### Patients and samples

A total of 753 patients including 512 HIV-1 and HCV co-infected patients were enrolled in a longitudinal study to investigate the response of HCV infection to IFN-α and ribavirin with simultaneous administration of antiretroviral therapy (ART) in the Eighth People’s Hospital, Guangzhou, China. The subjects were followed up to 240 weeks from the time of enrollment. In addition, 240 HCV RNA positive patients were recruited from Nanfang Hospital, Guangzhou, China to receive treatment with DAAs. Patient HCV121 has previously been reported to be infected with HCV and HPgV-2 [26]. Clinical information was obtained from hospital records. Written informed consents were obtained from all participants. This study was approved by the ethical committee of Guangzhou Eighth People’s Hospital (No.201816107) and Nanfang Hospital (NFEC-2017-046).

At different time points, peripheral blood mononuclear cells (PBMCs) were isolated from participants using Ficoll-Paque PLUS (Catalog#10248245, SWEDEN) immediately after blood collection. CD4^+^ and CD8^+^ T cell and B cell subsets were separated from PBMCs using MoFlo sorting flow cytometry with antibodies obtained from BD Biosciences, USA: FITC labelling of mouse anti-human CD4 antibody (# 555346); APC labelling of mouse anti-human CD19 antibody (#555415); and PE labelling of mouse anti-human CD8 antibody (#555086). Screening for liver fibrosis was performed by Fibroscan (Echosens, Paris, France) [27]. Percutaneous liver biopsies were performed with specimens obtained from patients HCV121 and JX18052, respectively, before treatment with DAAs and 12 weeks after termination of the treatment. All samples were stored at −80°C until they were analysed.

### Detection of anti-HPgV-2 antibody and HPgV-2 RNA

Enzyme-linked immunosorbent assay (ELISA) for detecting anti-HPgV-2, and reverse transcription–polymerase chain reaction (RT–PCR) for detection and quantification of HPgV-2 RNA from plasma have been reported previously [26]. To enhance the amplification specificity of HPgV-2 positive and negative-strand RNAs in PBMCs, we adapted a tagged primer approach described by Xiang et al. [28], in which a special primer that contains both an HPgV-2 specific sequence and a non-HPgV-2 tag sequence was used for cDNA synthesis during the reverse transcription reaction (Supplementary Table S1). To ensure the specific amplification from the above HPgV-2 cDNA, subsequent amplification of HPgV-2 cDNA used only the tag sequence primer and another HPgV-2 specific primer.

### Detection and quantification of HCV RNA and HIV-1 RNA

RT–PCR for detection of HCV RNA from plasma has been reported previously [26]. Quantification of virus RNA from plasma was performed using the Cobas Amplicor Hepatitis C Virus Test and HIV-1 Test (Roche, USA). For detection of HCV positive and negative RNAs in PBMCs, a strategy similar to that used for HPgV-2 RNA detection was utilized with the primers shown in Supplementary Table S1.

### Histological study

Liver tissue samples were fixed in formalin and embedded in paraffin. Sections of 4–6 µm thicknesses were stained with hematoxylin and eosin (H.E.) for general histopathological examination and with Masson’s trichrome reagent for fibrosis assessment. Modified hepatic inflammation activity (HIA) and fibrosis stage (S) were scored according to the Ishak classification [29]. The hepatic histomorphological analysis was performed independently by two different pathologists.

### Immunohistochemistry staining of HPgV-2 antigens in liver tissues

Antiserum (WG-03395) against the polypeptide P9 of HPgV-2 NS5A protein [10] was produced in rabbits and was purified by affinity chromatography at the Abclone company (Wuhan, China). The specificity of the anti-HPgV-2 antibody used in our study has been evaluated. The polyclonal antiserum only reacted with the peptide P9 of HPgV-2 NS5A, not the NS3 peptide P4 and the NS5A/B peptide P16 (supplemental Fig. S1A). Furthermore, the polyclonal anti-HPgV-2 antibody can inhibit the binding of HPgV-2 positive sera in the competitive inhibition studies (Fig. S1B). These results demonstrated the specificity of the polyclonal antibodies against HPgV-2 NS5A. Liver sections of 4–6 µm thicknesses were cut for immunohistochemical staining (IHC) by using anti-HPgV-2 as the primary antibody (1:2500 dilution) and biotinylated rat-anti-rabbit antibody as the secondary antibody (1:300 dilution, BD Biosciences, USA). Images were captured using an Olympus microscope (BX53, Tokyo, Japan) equipped with an Olympus camera (U-TV0.83XB, Japan).

### In situ hybridization for HCV and HPgV-2 RNA

In situ hybridization of viral RNA in liver slices was performed by RNAscope, as previously described [30], with specific probes for the positive-strands of HPgV-2 and HCV. Paraformaldehyde-fixed PBMCs from HPgV-2-infected and uninfected patients were analysed using fluorescent in situ hybridization (FISH) with specific probes for the positive and negative strands of HPgV-2 and HCV (Supplementary Table S2).

## Results

### HPgV-2 causes both persistent and resolved infection

**Figure 1.**
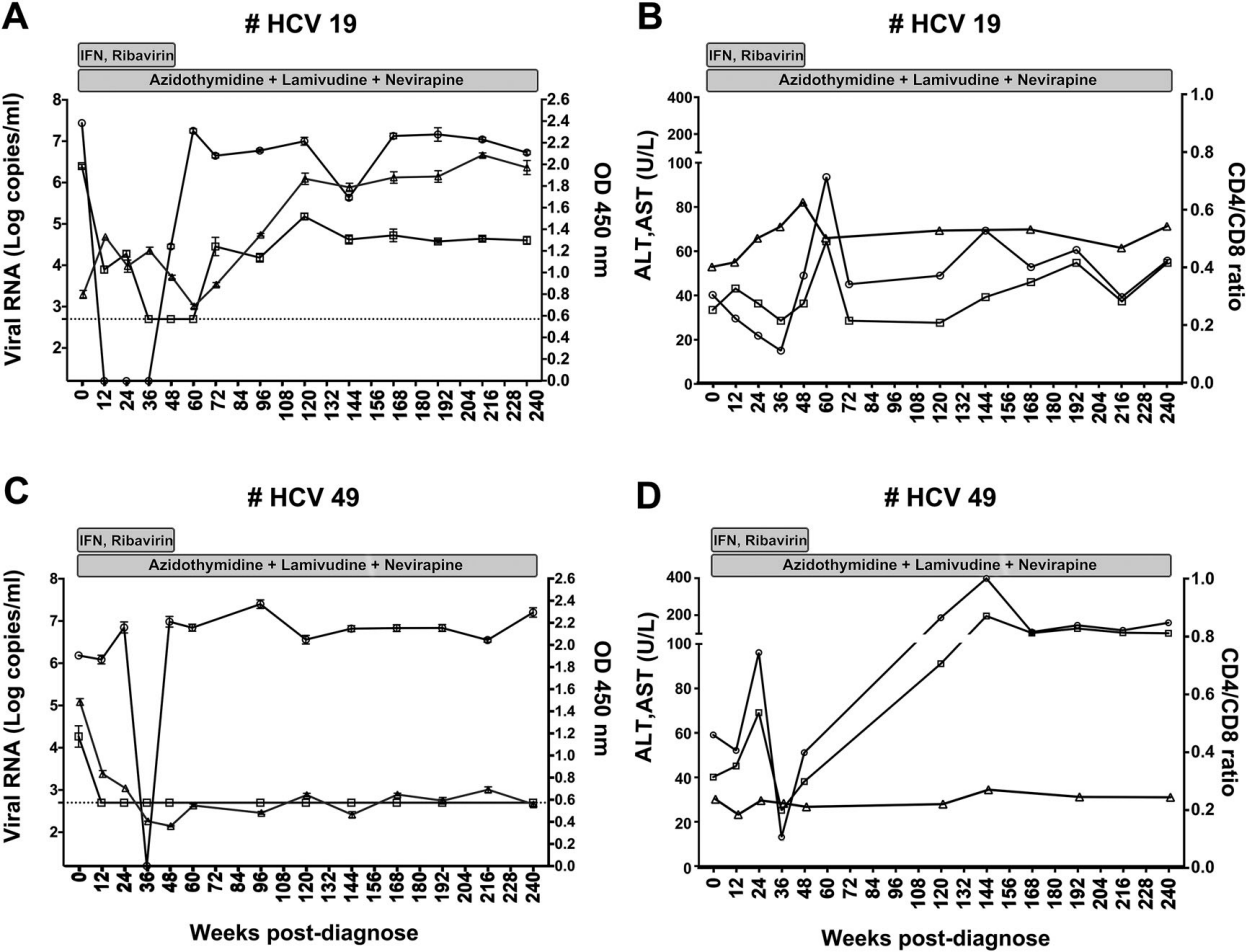
Persistent and resolved HPgV-2 infection. Two patients HCV19 (A, B) and HCV49 (C, D) from the HCV/HIV-1 co-infection cohort were followed for up to 240 weeks. Changes of viral RNA for HCV (circle, detection limit is 17 copies/ml by Cobas quantification) and HPgV-2 (square, detection limit is 750 copies/ml by RT-PCR) as well as anti-HPgV-2 antibody (triangle, cut-off value=0.2) were analysed and shown in the left panels. Levels of ALT (circle), AST (square) and CD4/CD8 ratio (triangle) were depicted in the panels to the right. The two patients received interferon and ribavirin therapy for 48 weeks and antiretroviral therapy as indicated in gray rectangles.

**Figure 2.**
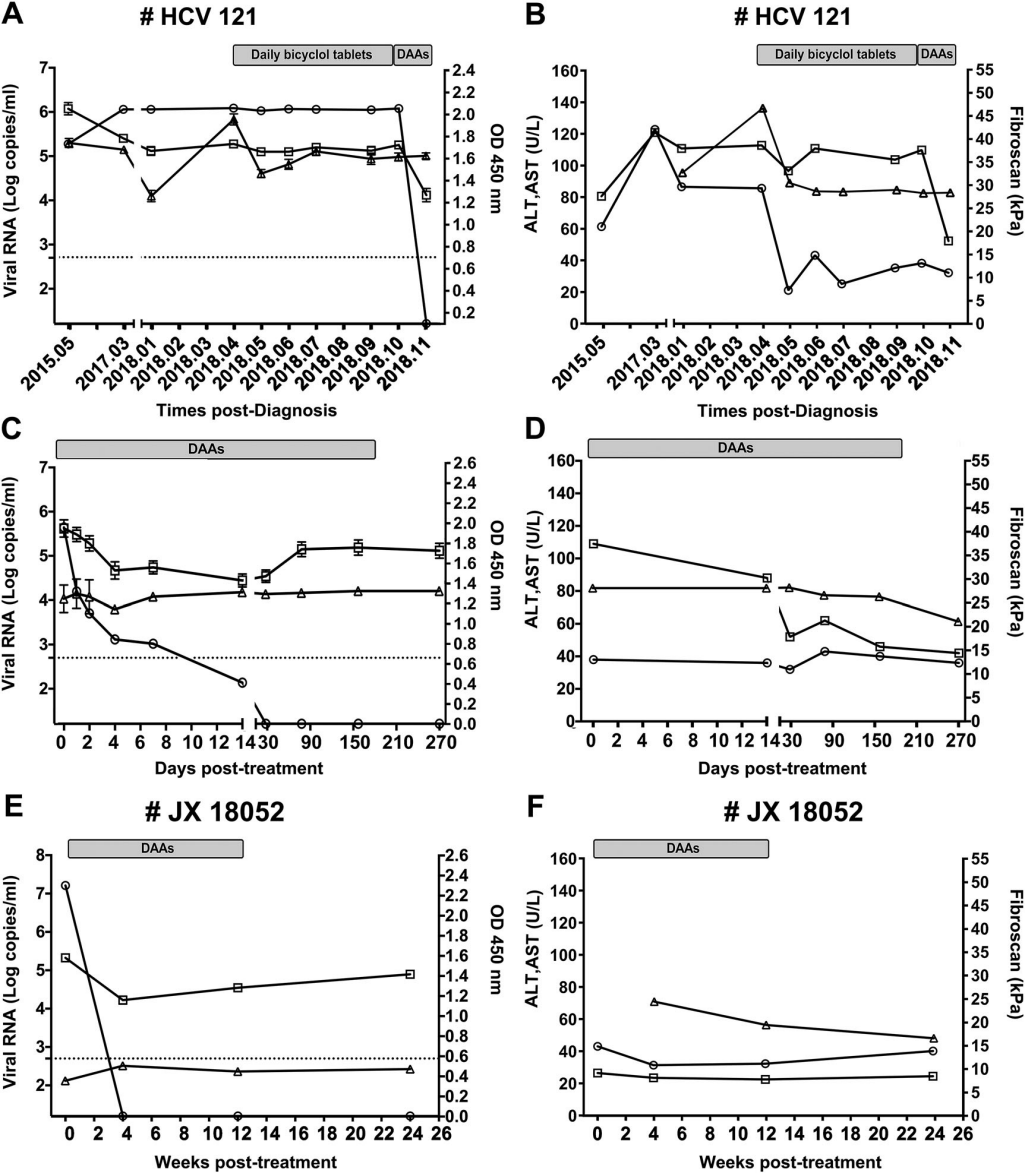
Clinical course of patients co-infected with HCV and HPgV-2. Changes in viral RNA for HCV (circle, detection limit is 17 copies/ml by Cobas quantification) and HPgV-2 (square, detection limit is 750 copies/ml by RT-PCR) as well as anti-HPgV-2 antibody (triangle, cut-off value=0.2) are presented in panels A, C, and E; Values for ALT (circle), AST (square) and Fibroscan score (triangle) are shown in panels B, D, and F. Patient HCV121 was followed from May 2015 to July 2019. During April and November 2018, bicyclol tablets were administered to improve liver function. Beginning in November 2018, DAAs (400 mg Sofosbuvir and 100 mg Velpatasvir) were administered for a period of 24 weeks. Patient JX18052 was co-infected with HCV and HPgV-2 and was treated with DAAs (Sofosbuvir + Daclatasvir) for 12 weeks. Both patients were followed up for another 12 weeks after termination of DAA treatment.

We identified 5 individuals with persistent HPgV-2 infection (Table 1). Patient HCV19 was co-infected with HCV and HIV-1 and was followed for 240 weeks (Figure 1(A)). He was given IFN-α and ribavirin during the first 48 weeks and anti-retroviral therapy (ART) throughout the follow-up period (Figure 1(A,B)). He displayed transient inhibition of HCV viremia followed by an increase of HCV RNA accompanied by an increase of alanine aminotransferase (ALT) and aspartate aminotransferase (AST), indicating virological failure for HCV treatment. Transient inhibition of HPgV-2 RNA followed by persistent HPgV-2 viremia and a high level of anti-HPgV-2 antibody were also observed in this patient (Figure 1(A)). Another patient, HCV121, was diagnosed as being co-infected with HCV and HPgV-2 since May 2015 and was persistently positive for HPgV-2 RNA and anti-HPgV-2 antibody (Figure 2(A)). These results indicated chronic and persistent HPgV-2 infection, which are defined as the presence of detectable viral replication for at least 6 months.

**Table 1.** Baseline characteristics of the patients co-infected with HCV and HPgV-2.

Patient ID	HCV RNA (copies/ml)	HCV genotype	HPgV-2 RNA (copies/ml)	AST (U/L) (15-40)	ALT (U/L) (9-50)	Fibroscan Scores (kPa)	Ultrasonic scan
HCV121	3.28 × 10^4^	3a	1.5 × 10^6^	80	61	NT	Liver cirrhosis, spleenomegaly
JX18052	1.63 × 10^7^	6a	4.46 × 10^5^	25	42	19.2	liver fibrosis, spleenomegaly
JX18145	1.02 × 10^7^	3a	1.07 × 10^5^	105	225	14.1	liver fibrosis
HCV19	2.94 × 10^7^	6a	2.59 × 10^6^	32	39	NT	NT
HCV49	1.60 × 10^6^	1a	4.00 × 10^4^	59	40	NT	NT

The viral loads of HCV and HPgV-2 were determined by quantitative RT-PCR. HCV genotyping was determined by PCR and Sanger sequencing based on partial Core-E1 region. The normal range of serum transaminase (AST, ALT) is indicated in brackets. Fibroscan score was measured by means of transient elastography (Echosens Corporation, Paris, France). NT, not tested; HCV, hepatitis C virus; HPgV-2, the second human pegivirus.

Interestingly, a resolved HPgV-2 infection was documented in patient HCV49, who is co-infected with HCV and HIV-1. HPgV-2 RNA decreased from 4.5 log_10_copies/ml at the beginning of the study to undetectable levels 12 weeks after treatment with IFN-α and ribavirin and remained negative up to 240 weeks post-treatment, the end of the study. In contrast, anti-HPgV-2 antibody was only weakly positive (Figure 1(C)). However, except for the initial transient inhibition of HCV replication as well as the decline of ALT and AST, persistent HCV viremia (Figure 1(C)) and abnormal ALT and AST levels (Figure 1(D)) were observed. In addition, HIV-1 RNA was undetectable post-ART (data not shown), and the ratio of CD4/CD8+ T cells increased during the follow-up period post-ART for the two patients HCV19 and HCV49 (Figure 1(B,D)).

### DAAs inhibit HCV replication, but not replication of HPgV-2

Patient HCV121 was initially found to be co-infected with HCV and HPgV-2 and also exhibited elevated ALT and AST values in May, 2015 [26]. He received a daily bicyclol tablet to improve his liver function from April to October 2018. Both ALT levels and fibroscan scores decreased after bicyclol treatment (Figure 2(B)). It is known that DAAs specifically target HCV polymerase and can clear HCV replication [31]. To determine the effect of treatment with DAAs on dual infection of HCV and HPgV-2, patient HCV121 was given DAAs (Sofosbuvir + Velpatasvir) in October 2018 (Figure 2(A)). HCV RNA levels gradually decreased and were undetectable at 28 days post-treatment with DAAs (Figure 2(C)). This decrease was accompanied by a reduction in AST to the reference level 14 days following administration of DAAs (Figure 2(D)). However, HPgV-2 RNA and anti-HPgV-2 antibody titers were maintained at relatively high levels, despite a slight decline in HPgV-2 RNA that was observed within one-week post-DAAs (Figure 2(C)).

We further identified two HPgV-2 RNA positive patients (JX18052 and JX18145) in a cohort of 240 HCV-infected patients (Table 1). Results similar to those obtained with patient HCV121 were observed in patient JX18052, who was treated with DAAs (Sofosbuvir plus Daclatasvir) for 12 weeks (Figure 2(E,F)). Taken together, these data clearly indicate that DAAs inhibit HCV, but not HPgV-2 replication. These results also suggest that HCV is not essential for replication of HPgV-2.

**Figure 3.**
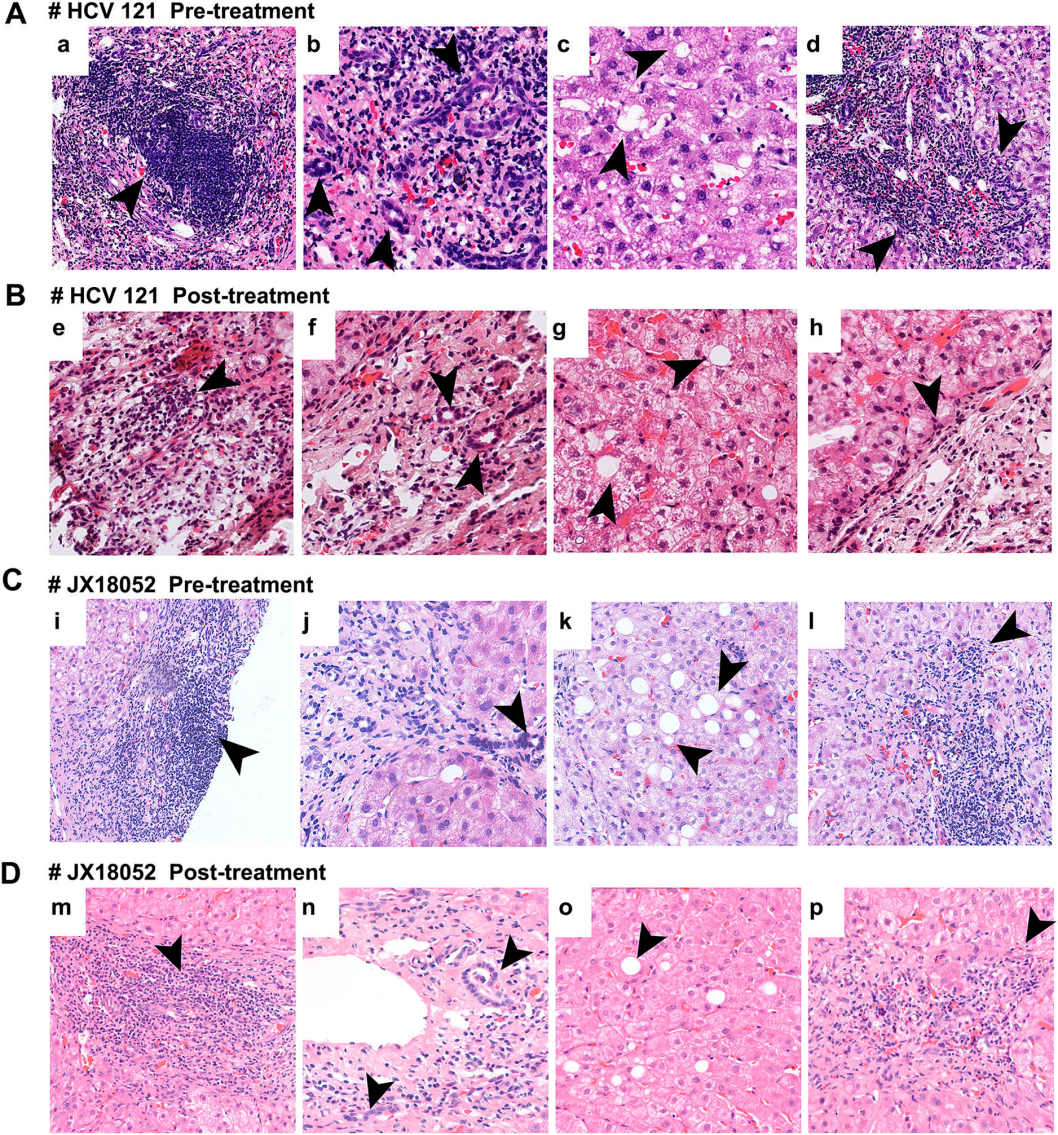
HE staining of liver slices showing representative pathological changes in patients HCV121 (A, B) and JX18052 (C, D) co-infected with HCV and HPgV-2. Before treatment (A, C), there was a large amount of lymphocyte infiltration in the portal tract and lymphoid follicles containing germinal centers (a, i); damaged bile ducts and hyperplasia of bile ducts in the portal tract (b, j); and macrovesicular steatosis (c, k). The interface was destroyed by lymphocyte infiltration and inflammation (d, l). After DAA treatment (B, D), there were fewer lymphocyte infiltrates in the portal tract (e, m); moderate bile duct hyperplasia in the portal tract (f, n); less macrovesicular steatosis in hepatocytes (g, o); and moderate interface inflammation (h, p).

Furthermore, liver biopsy samples from patient HCV121 were subjected to H.E. staining before and after 12 weeks of treatment (Figure 3(A,B); Supplementary Fig S2A, S2C). Interestingly, DAAs treatment in this case reduced liver inflammation and injuries and was accompanied by fewer lymphocyte infiltrations in the portal tract (Figure 3(A,E)), milder hyperplasia of ducts in the portal tract (Figure 3(B,F)), fewer macrovesicular steatosis of hepatocytes (Figure 3(C,G)), and milder interface inflammation (Figure 3(D,H)). Similar results were obtained for the HCV/HPgV-2 co-infected patient JX18052 (Figure 3(C,D); Supplementary Fig. S3A, S3C). Masson’s staining showed no significant change in the fibrosis stages of the two patients examined (Supplementary Fig. S2B, S2D; Supplementary Fig. S3B, S3D). These results were consistent with fibrosis stage (S) scores, although there was a slight difference between the two patients with respect to hepatic inflammation activity (HIA) (Supplementary Table S3). These results demonstrated that DAAs treatment led to an improvement in the extent of liver injuries. However, no specific pathological findings could be definitively related to HPgV-2 infection, suggesting that HPgV-2 infection is not associated with abnormal liver function.

### Tissue tropism of HPgV-2

**Figure 4.**
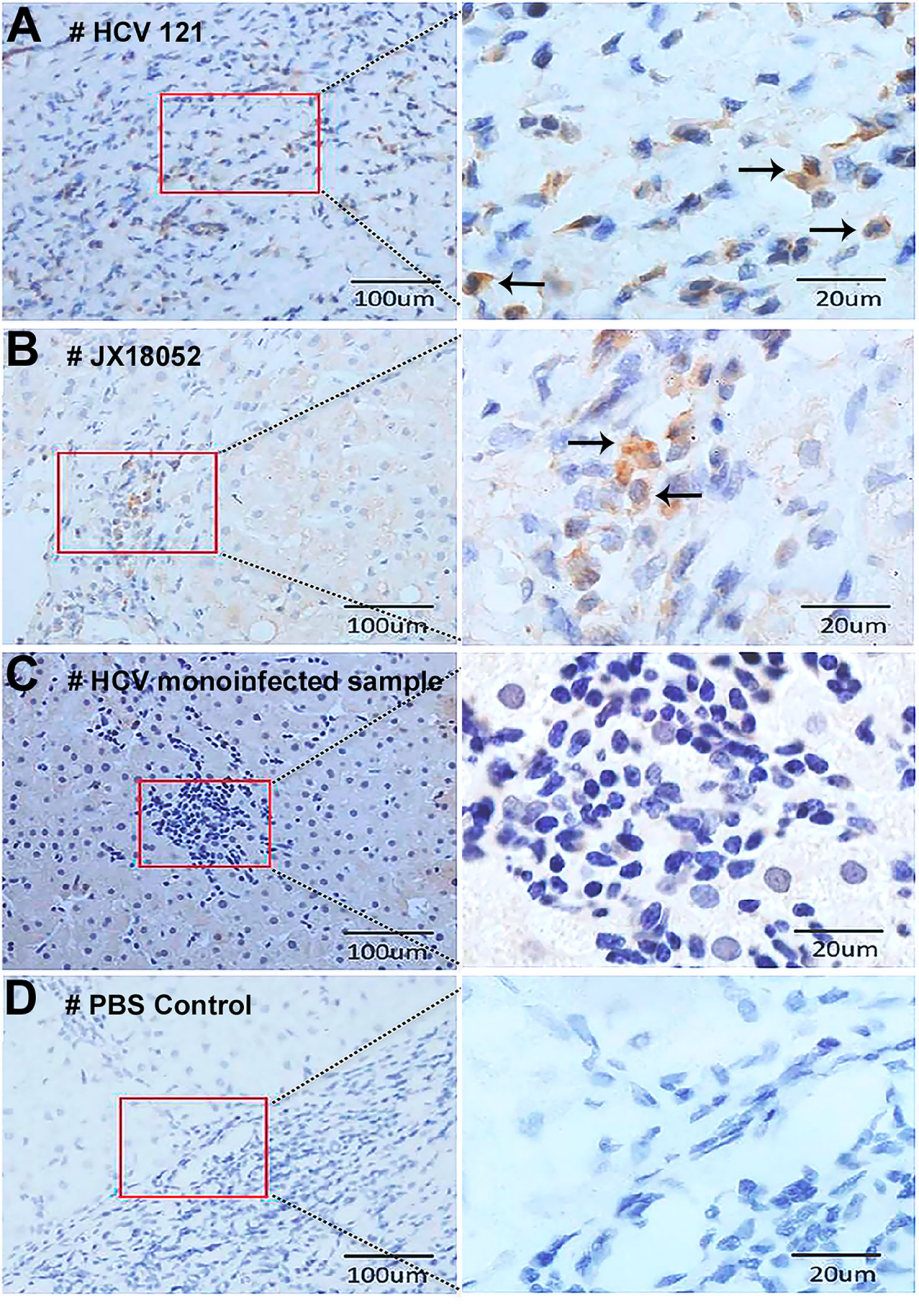
Detection of HPgV-2 antigen in liver tissues. Immunohistochemical (IHC) staining of liver biopsies was performed for HCV/HPgV-2 co-infected patients HCV121 (A) and JX18052 (B), using anti-HPgV-2 NS5A antibody as the primary antibody; HPgV-2 antigens were specifically stained brown in the infiltrative lymphocytes, but not in the hepatocytes. No specific signals were observed in the HCV positive, HPgV-2 negative patient (C). The negative control was stained using PBS to replace the primary antibody, anti-HPgV-2 (D).

Distribution of HPgV-2 antigen in the liver was analysed by using IHC. Interestingly, the antigen was found in infiltrative lymphocytes, but not in the hepatocytes present in the liver samples of HCV/HPgV-2 co-infected patients HCV121 (Figure 4(A)) and JX18052 (Figure 4(B)). No HPgV-2 antigen was observed in the liver slices of the HCV RNA positive, HPgV-2 negative, patient (Figure 4(C)). We further quantified the number and proportion of HPgV-2 infected cells in the liver. For each liver section, three fields were evaluated. The percentage of HPgV-2 NS5A positive cells was 11.4% (24/210) and 10.90% (18/165) for patient HCV121 and JX18052, respectively. However, staining of liver slices from HPgV-2 positive patients with anti-HPgV-2 antibody elicited a positive signal and as expected, no signal was detected when the liver slices were stained with a PBS control solution (Figure 4(D)).

**Figure 5.**
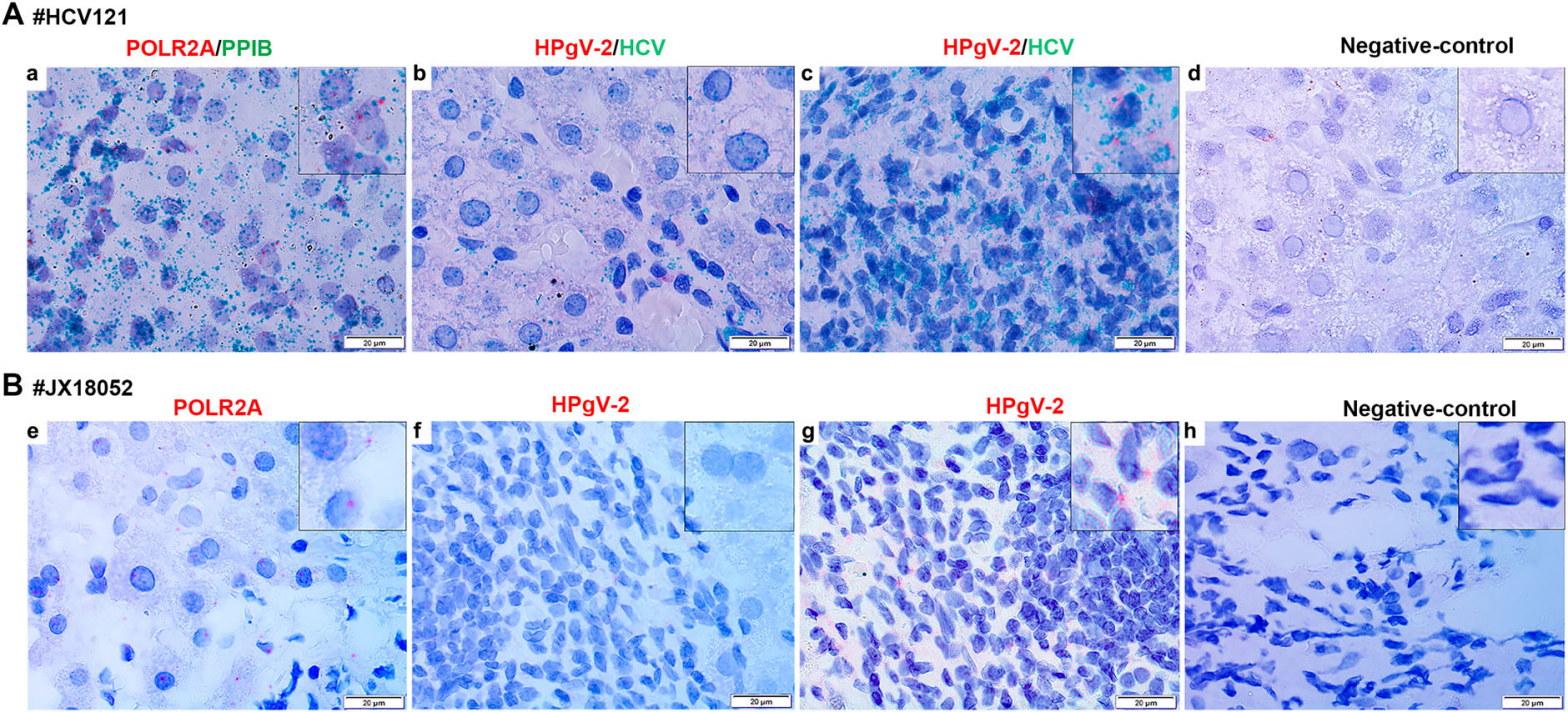
Detection of HPgV-2 and HCV RNA in liver tissues using RNAscope *in situ* hybridization. Liver slices were collected from HCV and HPgV-2 co-infected patients: patient HCV121 before DAA treatment (A) and patient JX18052 after DAA treatment (B). The specific probes for host gene POLR2A and PPIB were used as positive controls (a, e). For patient HCV121, HCV RNA (green) was detected in hepatocytes (b) and infiltrative lymphocytes (c), while HPgV-2 RNA (red) was only found in infiltrative lymphocytes (c). For patient JX18052, HPgV-2 RNA was detected in the infiltrative lymphocytes (g), but not in the hepatocytes (f). Non-specific probes were used as the negative controls (d, h). The size of the bar is 20 µm.

Furthermore, using RNAscope technology with specific probes of HCV and/or HPgV-2, HPgV-2, RNA signals were detected in the infiltrated lymphocytes in liver slices from patients HCV121 and JX18052, (Figure 5(C,G)), but not in their hepatocytes (Figure 5(B,F)). We quantified the percentage of HPgV-2 RNA positive cells in the liver sections, which was 5.45% (12/220) for patient HCV121 and 4.10% (10/245) for patient JX18052 whereas HCV RNAs were found in approximately 50% of the hepatocytes and infiltrated lymphocytes*.* These results indicate that unlike HCV, HPgV-2 seems to infect only lymphocytes, but not hepatocytes present in the liver samples.

**Figure 6.**
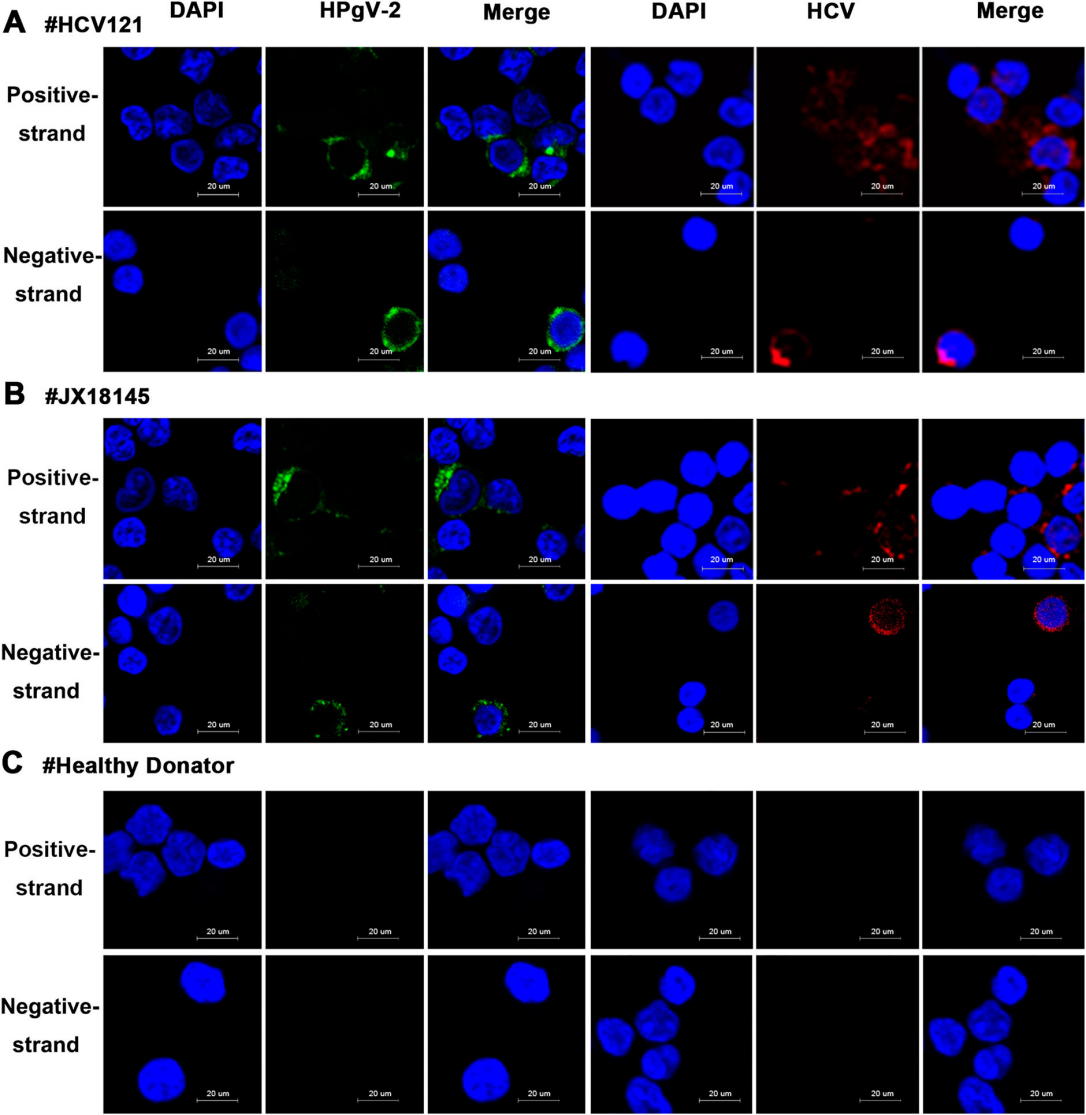
Detection of HPgV-2 and HCV in PBMCs using Fluorescent *in situ* Hybridization (FISH). Specific probes for the positive and negative-strand RNAs of HCV and HPgV-2 were end-labeled with TAMRA (red) and FAM (green), respectively. Before DAAs treatment, PBMCs from HCV and HPgV-2 co-infected patients HCV121 (A) and JX18145 (B) were hybridized with the labeled probes. (C) PBMCs from healthy volunteers were used as the negative control. The nucleus was stained with 1% DAPI (blue). The size of the bar is 20 µm.

**Figure 7.**
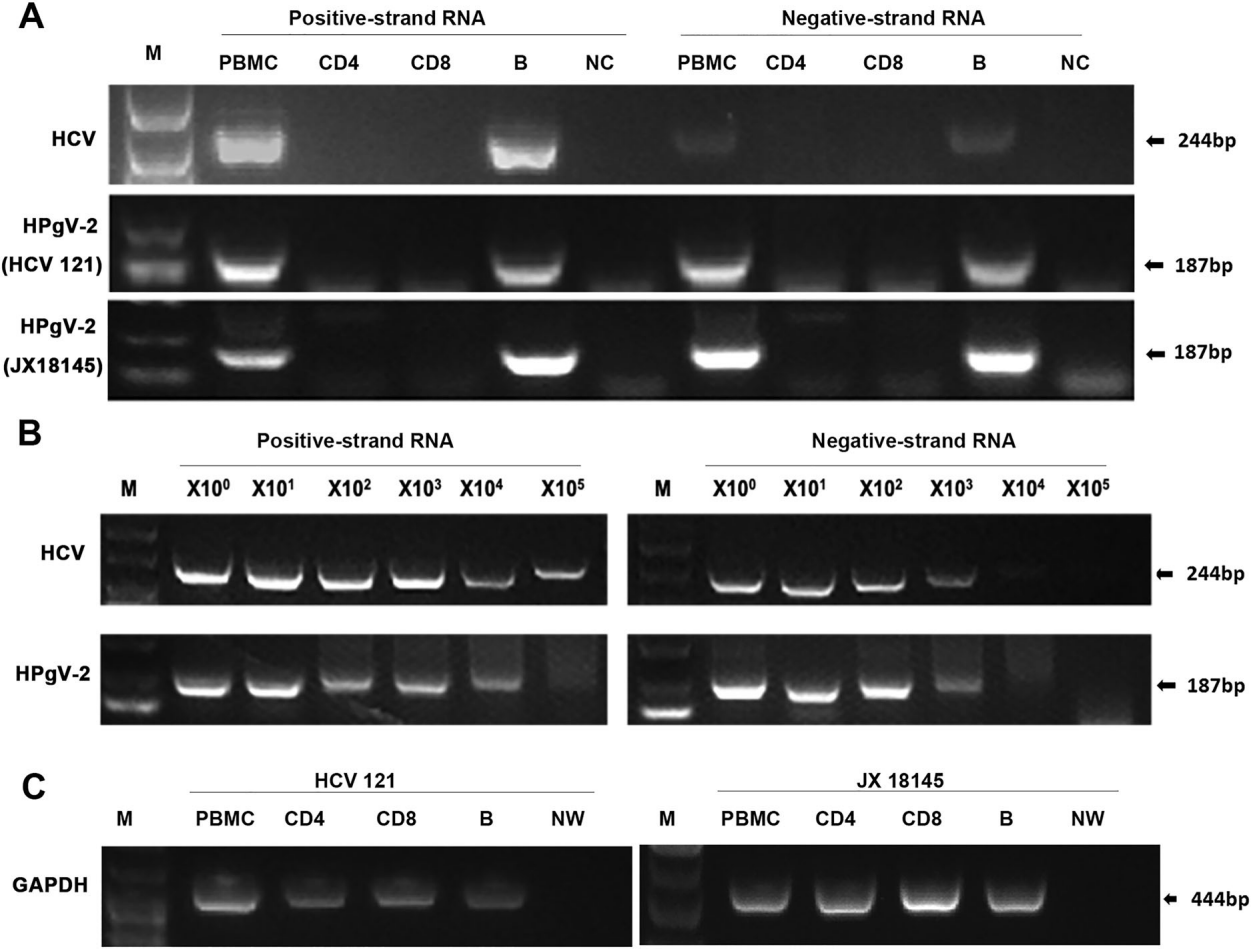
Amplification and detection of HPgV-2 and HCV positive- and negative-strand RNAs in PBMCs. CD4+ and CD8+ T lymphocytes as well as B cells were isolated with more than 90% purity using flow cytometry from HPgV-2 and HCV RNA positive patients HCV121 and JX18145. Total RNA was extracted from 2–4 × 10^6^ cells and amplified by RT-PCR for HCV and HPgV-2 (A). To measure virus replication efficiency within B cells, a semi-quantitative approach was used to quantify RNA levels by analysing 10-fold serial dilutions of the 1st round PCR products in the 2nd round PCR (B). The housekeeping gene GAPDH DNA was amplified in parallel and served as a control (C). M, Marker. NW, negative control using water; NC, negative control using PBMCs from healthy volunteer.

Further investigation using FISH technology demonstrated that both positive- and negative-strand HPgV-2 and HCV RNAs were present in PBMCs isolated from HPgV-2/HCV co-infected patients HCV121 (Figure 6(A)) and JX18145 (Figure 6(B)). No HPgV-2 RNA was found in the HPgV-2 negative patient (Figure 6(C)). For HCV 121, we found that the percentage of HPgV-2 and HCV positive-strand RNA positive cells was 9.26% (20/216) and 10.31% (23/223) respectively whereas 5.00% (12/240) and 6.78% (16/236) of the cells were positive for HPgV-2 and HCV negative-strand RNA, respectively. For JX18145, the percentage of HPgV-2 and HCV positive-strand RNA positive cells was 8.70% (17/207) and 10.43% (24/243), respectively whereas 5.77% (10/312) and 4.22% (7/166) of the cells were positive for HPgV-2 and HCV negative-strand RNA, respectively. To identify the lymphocyte subsets that supported HPgV-2 and HCV replication, CD4+ and CD8+ T cells as well as B cells were sorted by flow cytometry, using conditions that resulted in >90% purity of the isolated cells (data not shown). Both positive- and negative-strand HPgV-2 and HCV RNAs were detected in the B cells (Figure 7(A)). To measure virus replication efficiency within B cells, we took a semi-quantitative approach to quantify RNA levels by analysing 10-fold serial dilutions of the 1st round PCR products in the 2nd round PCR. The results showed that the level of HPgV-2 and HCV negative-strand RNA was at least 10- and 100-fold lower than that of HPgV-2 and HCV positive-strand RNA, respectively (Figure 7(B)). In contrast, the house-keeping gene, GAPDH, was detected in PBMCs, CD4, CD8, and B cells (Figure 7(C)).

In summary, the results of several types of analysis all indicate that HPgV-2 can infect and replicate in B cells and further demonstrate that HPgV-2 is a lymphotropic, but not a hepatotropic virus.

## Discussion

The tight association of the novel HPgV-2 virus with HCV and its potential clinical significance prompt us to probe the natural history of HPgV-2 infection and tissue tropism. In the current study, we provide new information regarding the natural history and replication of HPgV-2 infection. We demonstrate infection of HPgV-2 in PBMCs, specifically B-lymphocytes, but not hepatocytes (Figures 4–7). These results support the characterization of HPgV-2 as a lymphotropic, but not a hepatotropic virus. Furthermore, we show that DAAs that specifically target HCV polymerase and clear HCV infection in HCV and HPgV-2 dually-infected individuals are unable to inhibit HPgV-2 replication (Figure 2). To our knowledge, this is the first study to reveal that HPgV-2 persistent replication is independent of HCV viremia.

In view of our results and data from other studies regarding the independent replication of HPgV-2, an unresolved issue is how to explain the paradoxical finding that despite the epidemiological association of HPgV-2 and HCV infection, HPgV-2 replication seems not dependent on HCV viremia [5, 16, 25, 32]. Like HCV, HPgV-2 is a bloodborne virus [5, 10, 16]. Kandathil et al. have proposed that the association between HPgV-2 and HCV infection is due to the similarity of transmission routes of these two viruses and that multiple blood exposures could increase HPgV-2 infection [25]. However, our results as well as other studies indicate that these factors may not be sufficient to support the strong association between HPgV-2 and HCV infection [5, 10, 26]. For example, we found that the presence of HBV, another blood-borne virus, did not increase HPgV-2 infection in PWIDs [33]. Further investigation will be required to determine the factors affecting the high susceptibility of HPgV-2 in HCV-infected persons [11, 25, 26].

Of note, HPgV-2 and HPgV are in the same genus within the family of Flaviviridae. HPgV has also been shown to be a lymphotropic, rather than a hepatotropic virus [34], although it was initially thought to be hepatotropic and was originally called HGV [9]. Similar to HPgV-2, HPgV can replicate in PBMCs, although it appears to be pan-lymphotropic and can infect multiple mononuclear cells including CD4^+^ T cells, CD8^+^ T cells, B cells, monocytes, and NK-cells [35, 36]. Our results also show the infection and replication of HCV in B lymphocytes, which is consistent with earlier reports by Chen et al., who have determined the lymphotropism of HCV by using cellular protein B7.2 as co-receptor [37]. Wang et al. reported that the association of HCV with B cells occurs mainly through the interaction of complement receptor 2 (CD21) and the CD19/CD81 complex [38]. HCV is also associated with the risk of non-Hodgkin’s lymphoma (NHL) [39, 40]. These findings support B cells as the possible site where HCV and HPgV-2 co-localize and replicate.

At present, little is known about the mechanisms of HPgV-2 persistence. Lack of viral neutralization and virus-specific CD4^+^ T-cell function as well as high immune escape mutations are associated with HCV persistence [41]. In addition, the error-prone polymerases of HCV and HPgV result in extensive genetic diversity and many viral genome quasispecies [42, 43]. In contrast to HCV and HPgV, the genomic sequences of HPgV-2 are highly conserved and a hypervariable region is absent from the envelope proteins [5, 10, 13, 16, 26]. Moreover, antibodies directed against the envelope proteins are consistently detected in chronic HPgV-2-infected individuals [44]. Thus, neutralizing antibody escape may not be a strategy utilized by HPgV-2, which is supported by Forberg’s recent finding that the minimal geographic and temporal genetic diversity of HPgV-2 may be due to the minimal selective pressure on HPgV-2 to evolve [42].

In conclusion, this report is the first to indicate that HPgV-2 may be a lymphotropic, but not a hepatotropic virus, which in turn may explain the lack of HPgV-2 associated liver damage. Further research is needed to explore the association and interaction between HPgV-2 and HCV, the possible effect of HPgV-2 on immune modulation in various PBMCs, and its influence on viral persistence and disease association. We propose that a deeper understanding of HPgV-2 replication and immune modulation may provide critical insights into persistent infection of RNA viruses in humans.

## Supplementary Material

Supplemental Material

## References

[1] Simons JN, Pilot-Matias TJ, Leary TP, et al. Identification of two flavivirus-like genomes in the GB hepatitis agent. Proc. Natl. Acad. Sci. U.S.A.. 1995;92:3401–3405. doi: 10.1073/pnas.92.8.3401 7724574 PMC42174

[2] Baechlein C, Grundhoff A, Fischer N, et al. Pegivirus infection in domestic pigs, Germany. Emerging Infect. Dis.. 2016;22:1312–1314. doi: 10.3201/eid2207.160024 PMC4918184 27314228

[3] Chandriani S, Skewes-Cox P, Zhong W, et al. Identification of a previously undescribed divergent virus from the Flaviviridae family in an outbreak of equine serum hepatitis. Proc. Natl. Acad. Sci. U.S.A.. 2013;110:E1407–E1415. doi: 10.1073/pnas.1219217110 23509292 PMC3625295

[4] Firth C, Bhat M, Firth MA, et al. Detection of zoonotic pathogens and characterization of novel viruses carried by commensal Rattus norvegicus in New York City. mBio. 2014;5:e01933–14. doi: 10.1128/mBio.01933-14 25316698 PMC4205793

[5] Kapoor A, Kumar A, Simmonds P, et al. Virome analysis of transfusion recipients Revealsa novel human virus that shares genomic features with Hepaciviruses and Pegiviruses. mBio. 2015;6:e01466–15. doi: 10.1128/mBio.01466-15 26396247 PMC4600124

[6] Kapoor A, Simmonds P, Cullen JM, et al. Identification of a pegivirus (GB virus-like virus) that infects horses. J Virol. 2013;87:7185–7190. doi: 10.1128/JVI.00324-13 23596285 PMC3676142

[7] Quan PL, Firth C, Conte JM, et al. Bats are a major natural reservoir for hepaciviruses and pegiviruses. Proc. Natl. Acad. Sci. U.S.A.. 2013;110:8194–8199. doi: 10.1073/pnas.1303037110 23610427 PMC3657805

[8] Smith DB, Becher P, Bukh J, et al. Proposed update to the taxonomy of the genera Hepacivirus and Pegivirus within the Flaviviridae family. J Gen Virol. 2016;97:2894–2907. doi: 10.1099/jgv.0.000612 27692039 PMC5770844

[9] Linnen J, Wages J, Jr., Zhang-Keck ZY, et al. Molecular cloning and disease association of hepatitis G virus: a transfusion-transmissible agent. Science. 1996;271:505–508. doi: 10.1126/science.271.5248.505 8560265

[10] Berg MG, Lee D, Coller K, et al. Discovery of a novel human Pegivirus in blood associated with Hepatitis C virus co-infection. PLoS Pathog. 2015;11:e1005325. doi: 10.1371/journal.ppat.1005325 PMC4676677 26658760

[11] Anh NT, Hong NTT, Nhu LNT, et al. Detection and characterization of human Pegivirus 2, Vietnam. Emerging Infect. Dis.. 2018;24:2063–2067. doi: 10.3201/eid2411.180668 PMC6199981 30334714

[12] Rodgers MA, Holzmayer V, Vallari A, et al. Hepatitis C virus surveillance and identification of human pegivirus 2 in a large Cameroonian cohort. J. Viral Hepat.. 2018. 10.1111/jvh.12996 PMC7379692 30187640

[13] Wang HY, Wan ZW, Sun Q, et al. Second human pegivirus in Hepatitis C virus-infected and Hepatitis C virus/HIV-1-co-infected persons who inject drugs, China. Emerging Infect. Dis.. 2018;24:908–911. doi: 10.3201/eid2405.161162 PMC5938795 29664364

[14] Bijvand Y, Aghasadeghi MR, Sakhaee F, et al. First detection of human hepegivirus-1 (HHpgV-1) in Iranian patients with hemophilia. Sci Rep. 2018;8:5036. doi: 10.1038/s41598-018-23490-4 29568043 PMC5864744

[15] Lohmann V. Hepatitis C virus cell culture models: an encomium on basic research paving the road to therapy development. Med Microbiol Immunol. 2019;208:3–24. doi: 10.1007/s00430-018-0566-x 30298360

[16] Bonsall D, Gregory WF, Ip CLC, et al. Evaluation of Viremia Frequencies of a novel human Pegivirus by using bioinformatic screening and PCR. Emerging Infect. Dis.. 2016;22:671–678. doi: 10.3201/eid2204.151812 PMC4806942 26982117

[17] George SL, Xiang J, Stapleton JT. Clinical isolates of GB virus type C vary in their ability to persist and replicate in peripheral blood mononuclear cell cultures. Virology. 2003;316:191–201. doi: 10.1016/S0042-6822(03)00585-3 14644602

[18] Rydze RT, Bhattarai N, Stapleton JT. GB virus C infection is associated with a reduced rate of reactivation of latent HIV and protection against activation-induced T-cell death. Antivir. Ther. 2012;17:1271–1279. doi: 10.3851/IMP2309 22951385 PMC3709856

[19] Bhattarai N, Stapleton JT. GB virus C: the good boy virus? Trends Microbiol. 2012;20:124–130. doi: 10.1016/j.tim.2012.01.004 22325031 PMC3477489

[20] Chang CM, Stapleton JT, Klinzman D, et al. GBV-C infection and risk of NHL among U.S. adults. Cancer Res. 2014;74:5553–5560. doi: 10.1158/0008-5472.CAN-14-0209 25115299 PMC4184918

[21] Fama A, Xiang J, Link BK, et al. Human Pegivirus infection and lymphoma risk and prognosis: a North American study. Br J Haematol. 2018;182:644–653. doi: 10.1111/bjh.15416 29808922 PMC6108902

[22] Krajden M, Yu A, Braybrook H, et al. GBV-C/hepatitis G virus infection and non-Hodgkin lymphoma: a case control study. Int J Cancer. 2010;126:2885–2892. 19904755 10.1002/ijc.25035

[23] Fama A, Larson MC, Link BK, et al. Human Pegivirus infection and lymphoma risk: a systematic review and meta-analysis. Clinical infectious diseases: an official publication of the Infectious Diseases Society of America. 2019. 10.1093/cid/ciz940 PMC7442854 31671178

[24] Kennedy J, Pfankuche VM, Hoeltig D, et al. Genetic variability of porcine pegivirus in pigs from Europe and China and insights into tissue tropism. Sci Rep. 2019;9:8174. doi: 10.1038/s41598-019-44642-0 31160748 PMC6547670

[25] Kandathil AJ, Breitwieser FP, Sachithanandham J, et al. Presence of human Hepegivirus-1 in a cohort of people who inject drugs. Ann Intern Med. 2017;167:1–7. doi: 10.7326/M17-0085 28586923 PMC5721525

[26] Wang H, Wan Z, Xu R, et al. A novel human Pegivirus, HPgV-2 (HHpgV-1), is tightly associated with Hepatitis C virus (HCV) infection and HCV/human immunodeficiency virus Type 1 coinfection. Clin Infect Dis. 2018;66:29–35. doi: 10.1093/cid/cix748 29020289

[27] Machado MV, Cortezpinto H. Non-invasive diagnosis of non-alcoholic fatty liver disease. A critical appraisal. J Hepatol. 2013;58:1007–1019. doi: 10.1016/j.jhep.2012.11.021 23183525

[28] Xiang J, Wunschmann S, Schmidt W, et al. Full-length GB virus C (Hepatitis G virus) RNA transcripts are infectious in primary CD4-positive T cells. J Virol. 2000;74:9125–9133. doi: 10.1128/JVI.74.19.9125-9133.2000 10982359 PMC102111

[29] Wang Y, Rao H, Chi X, et al. Detection of residual HCV-RNA in patients who have achieved sustained virological response is associated with persistent histological abnormality. EBioMedicine. 2019;46:227–235. doi: 10.1016/j.ebiom.2019.07.043 31345785 PMC6711338

[30] Deleage C, Chan CN, Busman-Sahay K, et al. Next-generation in situ hybridization approaches to define and quantify HIV and SIV reservoirs in tissue microenvironments. Retrovirology. 2018;15:4. doi: 10.1186/s12977-017-0387-9 29316956 PMC5761108

[31] Feld JJ, Jacobson IM, Hezode C, et al. Sofosbuvir and Velpatasvir for HCV Genotype 1, 2, 4, 5, and 6 infection. N Engl J Med. 2015;373:2599–2607. doi: 10.1056/NEJMoa1512610 26571066

[32] Coller KE, Bruce V, Cassidy M, et al. Chronic human Pegivirus 2 without Hepatitis C virus co-infection. Emerging Infect. Dis.. 2020;26:265–272. doi: 10.3201/eid2602.190434 PMC6986836 31961294

[33] Shui J, Liu W, Liang Y, et al. Infection of human Pegivirus 2 (HPgV-2) is associated with Hepatitis C virus but not Hepatitis B virus infection in people who inject drugs. J Gen Virol. 2019;100:968–974. doi: 10.1099/jgv.0.001266 31090532

[34] Stapleton JT, Williams CF, Xiang J. GB virus type C: a beneficial infection? J Clin Microbiol. 2004;42:3915–3919. doi: 10.1128/JCM.42.9.3915-3919.2004 15364968 PMC516331

[35] Chivero ET, Bhattarai N, Rydze RT, et al. Human Pegivirus RNA is found in multiple blood mononuclear cells in vivo and serum-derived viral RNA-containing particles are infectious in vitro. J Gen Virol. 2014;95:1307–1319. doi: 10.1099/vir.0.063016-0 24668525 PMC4027039

[36] George SL, Varmaz D, Stapleton JT. GB virus C replicates in primary T and B lymphocytes. J Infect Dis. 2006;193:451–454. doi: 10.1086/499435 16388494

[37] Chen CL, Huang JY, Wang CH, et al. Hepatitis C virus has a genetically determined lymphotropism through co-receptor B7.2. Nat Commun. 2017;8:13882. doi: 10.1038/ncomms13882 28067225 PMC5227552

[38] Wang RY, Bare P, De Giorgi V, et al. Preferential association of hepatitis C virus with CD19(+) B cells is mediated by complement system. Hepatology. 2016;64:1900–1910. doi: 10.1002/hep.28842 27641977 PMC5115962

[39] Engels EA, Chatterjee N, Cerhan JR, et al. Hepatitis C virus infection and non-Hodgkin lymphoma: results of the NCI-SEER multi-center case-control study. Int J Cancer. 2004;111:76–80. doi: 10.1002/ijc.20021 15185346

[40] Giordano TP, Henderson L, Landgren O, et al. Risk of non-Hodgkin lymphoma and lymphoproliferative precursor diseases in US veterans with hepatitis C virus. Jama. 2007;297:2010–2017. doi: 10.1001/jama.297.18.2010 17488966

[41] Rehermann B. Pathogenesis of chronic viral hepatitis: differential roles of T cells and NK cells. Nat Med. 2013;19:859–868. doi: 10.1038/nm.3251 23836236 PMC4482132

[42] Forberg K, Rodgers MA, Dawson GJ, et al. Human Pegivirus 2 exhibits minimal geographic and temporal genetic diversity. Virology. 2020;539:69–79. doi: 10.1016/j.virol.2019.10.012 31689572

[43] Simmonds P. Variability of hepatitis C virus. Hepatology. 1995;21:570–583. doi: 10.1002/hep.1840210243 7531173 PMC7165699

[44] Coller KE, Berg MG, Frankel M, et al. Antibodies to the novel human Pegivirus 2 are associated with active and resolved infections. J Clin Microbiol. 2016;54:2023–2030. doi: 10.1128/JCM.00515-16 27225404 PMC4963515

