# Supplementary material for: Evidence that the second human pegivirus (HPgV-2) is primarily a lymphotropic virus and can replicate independent of HCV replication
Source: Emerg Microbes Infect. 2020 Feb 26;9(1):485–95. doi: 10.1080/22221751.2020.1730247 (PMC7054972; doi:10.1080/22221751.2020.1730247)
Supplement: Supplemental Material [file TEMI_A_1730247_SM8205.zip › Supplementary Tables.docx]

Supplementary Table S1: Primers for amplifying negative and positive RNA of HPgV-2 and HCV.

| Genome | Primers | | | Sequence (5’- 3’) | Position |
| --- | --- | --- | --- | --- | --- |
| HPgV-2 Negative RNA | Reverse transcription | | | **TCATGGTGGCGAATAA**CAAGGGCCTAGTAGGACGTGTGACA | 79-99 |
|  | First Round PCR | | Forward | **TCATGGTGGCGAATAA** |  |
|  |  |  | Reverse | GCCACCCTATCAGGCTGTACGGAGT | 266-290 |
|  | Second Round PCR | | Forward | **TCATGGTGGCGAATAA** |  |
|  |  |  | Reverse | GCCGCCACCCTATCAGGCTGTA | 272-293 |
| HPgV-2 Positive RNA | Reverse transcription | | | **TCATGGTGGCGAATAA**GCCGCCACCCTATCAGGCTGTA | 272-293 |
|  | First Round PCR | | Forward | CAAGGGCCTAGTAGGACGTGTGACA | 79-99 |
|  |  |  | Reverse | **TCATGGTGGCGAATAA** |  |
|  | Second Round PCR | | Forward | GCCTAGTAGGACGTGTGACAATGCC | 80-104 |
|  |  |  | Reverse | **TCATGGTGGCGAATAA** |  |
| HCV Negative RNA | Reverse transcription | | | **TCATGGTGGCGAATAA** ACTGTCTTCACGCAGAAAGCGTCTAGCCAT | 56-85 |
|  | First Round PCR | | Forward | **TCATGGTGGCGAATAA** |  |
|  |  |  | Reverse | CGAGACCTCCCGGGGCACTCGCAAGCACCC | 298-327 |
|  | Second Round PCR | | Forward | **TCATGGTGGCGAATAA** |  |
|  |  |  | Reverse | TCCCGGGCACTCGCAAGCACCCTATCAGG | 291-319 |
| HCV Positive RNA | Reverse transcription | | | **TCATGGTGGCGAATAA**CGAGACCTCCCGGGGCACTCGCAAGCACCC | 298-327 |
|  | First Round PCR | Forward | | ACTGTCTTCACGCAGAAAGCGTCTAGCCAT | 56-85 |
|  |  | Reverse | | **TCATGGTGGCGAATAA** |  |
|  | Second Round PCR | Forward | | ACGCAGAAAGCGTCTAGCCTAGGCGTTAGT | 65-94 |
|  |  | Reverse | | **TCATGGTGGCGAATAA** |  |

Reference sequence KX528230.1 for HPgV-2 and [MG406988.1](https://www.ncbi.nlm.nih.gov/nucleotide/MG406988.1?report=genbank&log$=nuclalign&blast_rank=1&RID=JVTNZ253014) for HCV. Bold sequence, none-HCV and –HPgV-2 tag sequence.

Supplementary Table S2: Probes for detecting negative and positive RNAs of HPgV-2 and HCV used in fluorescent in situ hybridization (FISH).

| Genome | Probes for Negative-strand RNA (5’- 3’) | Probes for Positive-strand RNA (5’- 3’) | Position | Label |
| --- | --- | --- | --- | --- |
| HPgV-2* | AACTACCTGGTCTTCTGTGGCAAATGTTTGTGGGCTT | AAGCCCACAAACATTTGCCACAGAAGACCAGGTAGTT | 950-986 | FAM |
|  | ACAACTATCATACCACAGAACTGTCGCAACTCTACGGCTGA | AGCCGTAGAGTTGCGACAGTTCTGTGGTATGATAGTTGT | 1381-1421 |  |
|  | GCGTTCTGCTTCACTTCTGGTACTTGTGCTACTTTCAATGA | TCATTGAAAGTAGCACAAGTACCAGAAGTGAAGCAGAACGC | 2185-2225 |  |
|  | TCTTGAGGTTTGTGCGGACATCTCTTGGTTGGTGGAATT-3’ | AATTCCACCAACCAAGAGATGTCCGCACAAACCTCAA | 2877-2915 |  |
|  | TCTTTCTGAGGTCGTGGAAGATACAATGACTTTAGTGTGTGGACA | TGTCCACACACTAAAGTCATTGTATCTTCCACGACCTCAGAAA | 3435-3479 |  |
|  | AATGGAGGGTTGGTGTGTGGCAGAGTGGAGAATGAAG | CTTCATTCTCCACTCTGCCACACACCAACCCTCCATT | 3901-3937 |  |
|  | TCGTAGTCATCTGCGACGAGTGCCACGACACATCATCTA | TAGATGATGTGTCGTGGCACTCGTCGCAGATGACTAC | 4430-4468 |  |
|  | CCAGATGATAGCATCAAGTGGAAGAGCCTACTCAACAACACA | TGTGTTGTTGAGTAGGCTCTTCCACTTGATGCTATCATCTGG | 5305-5346 |  |
|  | TCCATTCAATGCCTTTGCTCAACATCTGACCCAATACTCA | TGAGTATTGGGTCAGATGTTGAGCAAAGGCATTGAATGGA | 6544-6583 |  |
|  | TCCCGAGACAAGTCAACAAGGAAGCCACCACGATTCATC | GATGAATCGTGGTGGCTTCCTTGTTGACTTGTCTCGG | 8170-8208 |  |
|  | GCCAGTGCTATACTCGCTCTGCTTTGCTTGTAAATCCTAAATC | GATTTAGGATTTACAAGCAAAGCAGAGCGAGTATAGCACTGGC | 9415-9457 |  |
| HCV^#^ | CCTTGTGGTACTGCCTGATAGGGTGCTTGCGAGTGCCC | GGCACTCGCAAGCACCCTATCAGGCAGTACCACAAGG | 278-314 | TAMRA |
|  | CAAAGAAAAACCAAAAGAAACACCATCCGTCGCCCACA | TGTGGGCGACGGATGGTGTTTCTTTTGGTTTTTCTTTG | 362-398 |  |
|  | GGACAGATCGTTGGTGGAGTATACGTGTTGCCGCGCAG | CTGCGCGGCAACACGTATACTCCACCAACGATCTGTC | 422-458 |  |
|  | GATCTCGCAATTTGGGTAAAGTCATCGATCCCCTTCCC | GGGAAGGGGATCGATGACTTTACCCAAATTGCGAGATC | 684-720 |  |
|  | AAATTGCACGACCATCATGGCGAAGAACGAGGTGTTT | AAACACCTCGTTCTTCGCCATGATGGTCGTGCAATTT | 8027-8064 |  |
|  | CGTGTCTGTGAGAAACGCGCCCTATATGACGTGATACA | GTATCACGTCATATAGGGCGCGTTTCTCACAGACACG | 8132-8168 |  |
|  | CCTACCAGCTTCGGCAACACAATCACTTGTTACATCAAGG | CCTTGATGTAACAAGTGATTGTGTTGCCGAAGCTGGTAGG | 8486-8524 |  |
|  | GTGGTGGCTGAGAGTGATGGCGTCGATGAGGATAGAGCA | CTCTATCCTCATCGACGCCATCACTCTCAGCCACCAC | 8594-8631 |  |
|  | GATGAGGATAGAGCAGCCCTGAGAGCTTTCACGGAGG | CCTCCGTGAAAGCTCTCAGGGCTGCTCTATCCTCATC | 8618-8653 |  |

*Reference sequence KX528230; #Reference sequence KC844041; carboxytetramethylrhodamine: TAMRA; carboxyfluorescein: FAM

Supplementary Table S3. Semi-quantitative histological evaluation for patients HCV 121 and JX 18052 pre- and post-DAAs treatment.

| Patients | HAI score* | | S score^#^ | |
| --- | --- | --- | --- | --- |
|  | Pre-treatment | Post-treatment | Pre-treatment | Post-treatment |
| HCV 121 | 17 | 12 | 6 | 6 |
| JX 18052 | 14 | 12 | 4 | 4 |

Liver biopsies were evaluated and staged independently by two pathologists, according to the Ishak classification. * The maximum possible score for HAI is 18. #The maximum possible score for S score is 6. HAI score, Hepatic inflammation activity score; S score, fibrosis stage score.
