# Supplementary figures and images for: Evidence that the second human pegivirus (HPgV-2) is primarily a lymphotropic virus and can replicate independent of HCV replication
Source: Emerg Microbes Infect. 2020 Feb 26;9(1):485–95. doi: 10.1080/22221751.2020.1730247 (PMC7054972; doi:10.1080/22221751.2020.1730247)

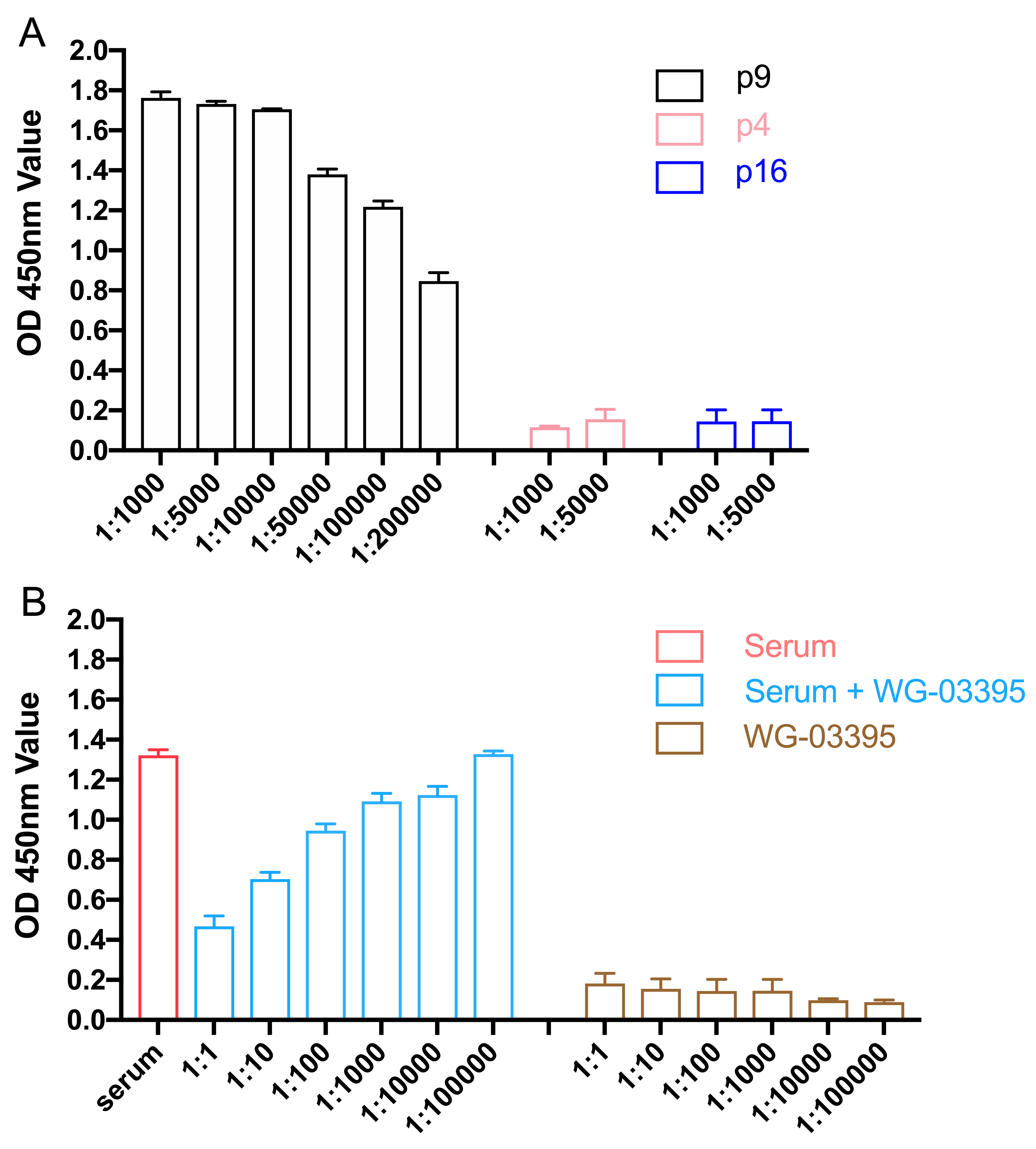

Supplement: Supplemental Material [file TEMI_A_1730247_SM8205.zip › Fig_S1_of_TEMI_2019_0744.R1_final.tiff]

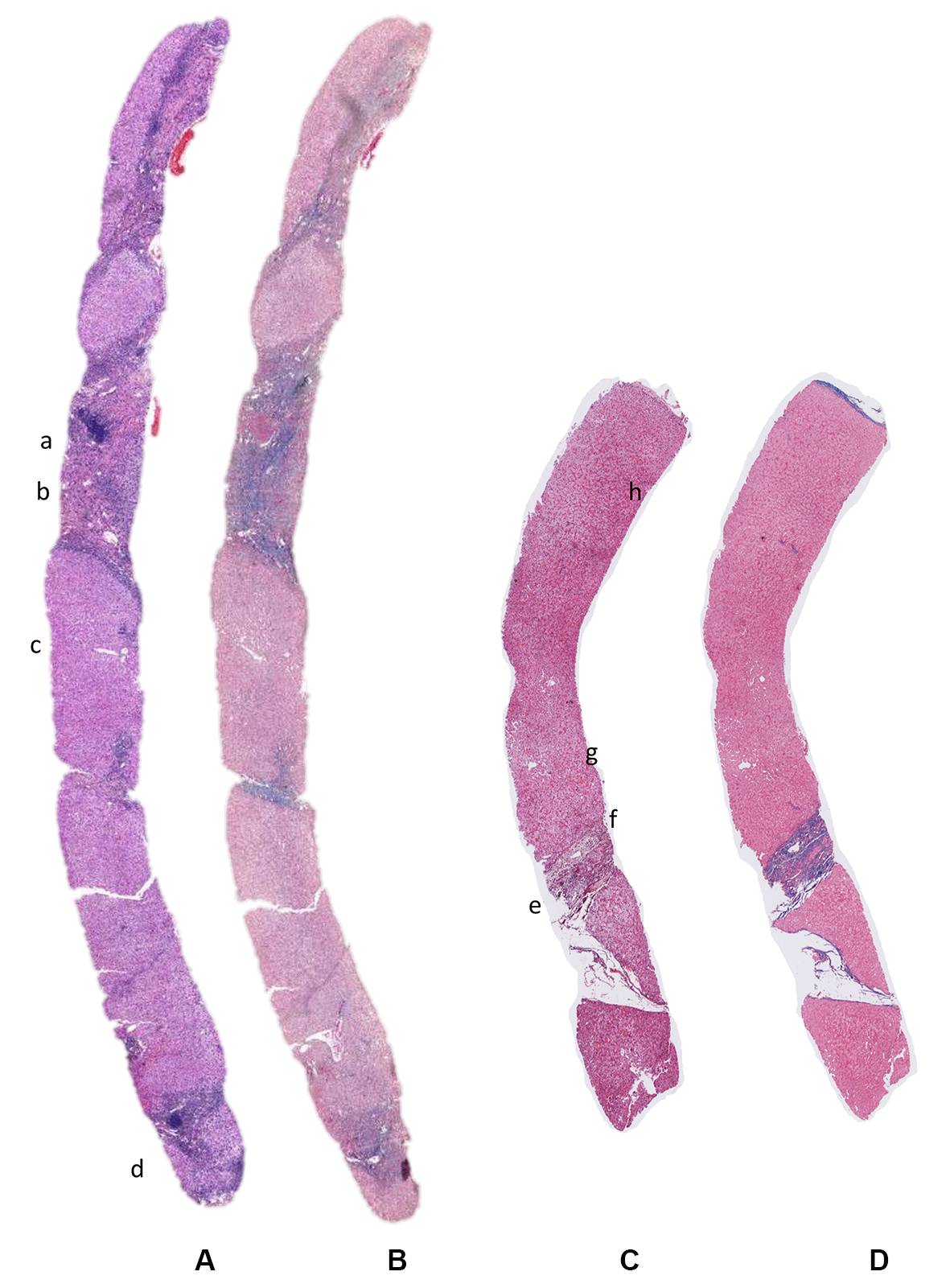

Supplement: Supplemental Material [file TEMI_A_1730247_SM8205.zip › Supplementary Figure S2.tif]

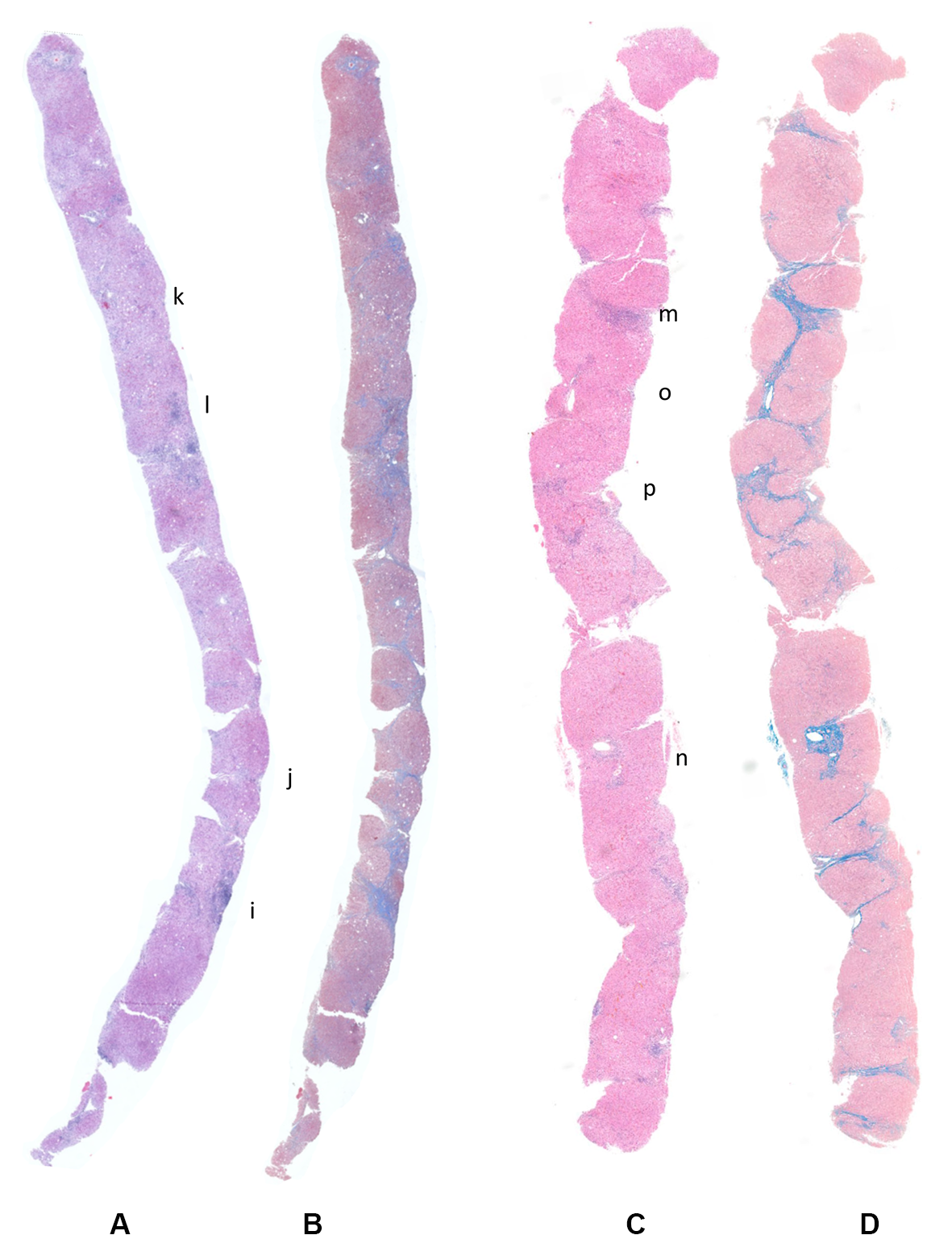

Supplement: Supplemental Material [file TEMI_A_1730247_SM8205.zip › Supplementary Figure S3.tif]
